# The COVID-19 Vaccine Is Here—Now Who Is Willing to Get It?

**DOI:** 10.3390/vaccines9040339

**Published:** 2021-04-01

**Authors:** William D. S. Killgore, Sara A. Cloonan, Emily C. Taylor, Natalie S. Dailey

**Affiliations:** Department of Psychiatry, College of Medicine, University of Arizona, Tucson, AZ 85724, USA; scloonan@psychiatry.arizona.edu (S.A.C.); emilytaylor@psychiatry.arizona.edu (E.C.T.); ndailey@psychiatry.arizona.edu (N.S.D.)

**Keywords:** vaccine hesitancy, COVID-19, political worldview

## Abstract

The U.S. vaccine campaign against COVID-19 began in December 2020, but many individuals seem reluctant to get vaccinated. During the first week of the vaccination campaign, we collected data from 1017 individuals with an online survey to identify factors that were associated with willingness to get the vaccine once it is available. Most participants (55.3%) were willing to get the vaccine, although 46.2% also expressed some fear of the vaccine. Political ideology was by far the most consistent predictor of both willingness to be vaccinated and fear of the vaccine, followed by participant sex, education level, income, and race/ethnicity. Our findings suggest that, for the vaccine campaign to be broadly supported and successful, it will be important for frontline healthcare workers to discuss the role of inoculation for COVID-19 in a manner consistent with each individual patient’s political and sociological worldview.

## 1. Introduction

In just a single year, the virus that causes COVID-19 emerged on the scene and infected approximately 99 million people worldwide, with over 400,000 deaths recorded in the U.S. alone. Remarkably, several vaccines were rapidly developed, rigorously tested, and fast-tracked for approval in well under a year, with the first doses administered in the U.S. on 14 December 2020. A primary goal of the immunization process has been to vaccinate enough of the population to reach a state of “herd immunity” to minimize viral transmission [1]. To accomplish this goal, the public needs to be well-informed and collectively supportive of the inoculation effort. Several factors, including the extraordinarily rapid pace of vaccine development, its fast-tracked approval, and politicization of pandemic response efforts, have fueled public skepticism about the potential safety and effectiveness of the newly developed vaccines [2]. Understanding the causes of vaccine hesitancy and ways to foster confidence among the public are vital to a successful inoculation effort [3,4]. The national response to the vaccination effort in the U.S. is likely to have major long-term consequences for public health and the economy. As this issue is so vital to public health, we assessed demographic, attitudinal, and psychiatric factors affecting people’s fear of, and their willingness to receive, the COVID-19 vaccines during the days when they first became available in the U.S.

## 2. Methods

### 2.1. Participants

Between 10–15 December 2020, the days encompassing the first release of the COVID-19 vaccines, a total of 1062 adults were initially recruited to participate in an online survey about COVID-19. Numerous safeguards were included to maximize the quality of the data. The survey was open only to English speaking individuals residing in the U.S. Of these, 27 potential participants (2.5%) were screened out due to failing a brief 6th grade-level reading screener. Thus, 1035 participants were enrolled. Of these, 1017 (98.3%) provided usable data on the questions of interest and passed all multiple embedded attention check items within the survey and are considered as the final sample for this analysis (see Table 1). These participants (58.4% female; 75.7% White) ranged in age from 18 to 91 years (Median age = 34; *Mean* = 37.0, *SD* = 12.2). While the proportion of females in the sample was slightly larger than that of the U.S. population, the proportion of White participants did not differ significantly from that of the 2019 U.S. Census. The median age in the present sample was about 4.4 years younger than the population median according to the U.S. Census. All participants completed written informed consent prior to enrollment and the protocol for this study was reviewed and approved by the Institutional Review Board (IRB) of the University of Arizona (protocol #2004536781).

### 2.2. Recruitment

Recruitment was accomplished via the Amazon Mechanical Turk (MTurk) online crowdsourcing platform [5], which reportedly has upwards of 250,000 members available in the U.S., and high turnover in new participants, providing an effective tool for recruiting naïve participants for certain types of survey research [6]. The MTurk platform is widely used by researchers and has been shown to provide data that are as valid and reliable as that obtained in in-person laboratory studies [7] and tend to provide higher quality data than that obtained from typical undergraduate samples [8], although concerns about data quality and generalizability of MTurk data have been raised by a number of authors [9,10]. Evidence suggests that the validity of MTurk responses can be enhanced by employing screening questions to eliminate problematic participants [7]. Therefore, to enhance data quality, we included an initial reading screener prior to the survey that prevented access to individuals who could not comprehend English at a 6th grade reading level. Poorly performing individuals and automated robotic response generators (i.e., “bots”) were prevented from entering the survey by a Captcha task administered prior to the informed consent document, as well as a “Bot Detection” algorithm incorporated by MTurk that flags suspicious responses, and an “Email Scan Roadblock” that prevents security scanners from starting a survey when they test the survey link, which is how most “bots” tend to get through. All U.S. individuals registered as TurkPrime members on the MTurk platform were sent an email message indicating that they might be eligible to complete an online survey regarding COVID-19. Participant location (based on IP address) was distributed broadly throughout the U.S. and included individuals from 48 U.S. states and the District of Columbia (i.e., responses from Vermont and Wyoming were not obtained). The proportion of participants from each state closely matched that of the 2019 U.S. Census, with all sampled state proportions differing from the census data by less than 2 percentage points, except for California (underrepresented by 3.5 percentage points) and Texas (underrepresented by 2.2 percentage points). The intraclass correlation coefficient between the sample proportions and the census data was quite high (ICC = 0.93, *p* < 0.0001), suggesting that these data are likely representative of the state-wise distribution of the larger population.

### 2.3. Materials

The survey included questions about demographics (age, sex, ethnicity, formal education, and annual income) and political ideology (7-point Likert scale ranging from “strongly liberal” to “strongly conservative”). Participants also reported whether they were “considered to be in a ‘high-risk’ group for COVID-19 (i.e., elderly, chronic lung disease, immunocompromised, BMI > 39, diabetes, liver disease, or other underlying health condition)?” (yes/no) and whether they had been diagnosed with COVID-19 (yes/no). Participants also completed brief assessments of depression (Patient Health Questionnaire-9; PHQ-9) [11], anxiety (Generalized Anxiety Disorder scale-7; GAD-7) [12], posttraumatic stress disorder (Primary Care Posttraumatic Stress Disorder; PC-PTSD) [13], and psychological resilience (Connor-Davidson Resilience Scale; CD-RISC) [14]. Finally, all participants provided responses to the following two questions on a 7-point Likert scale from “totally disagree” to “totally agree”, including: (Item 1) “I am afraid to the get the new COVID-19 vaccine” and (Item 2) “I will get the new COVID-19 vaccine as soon as it is offered to me.”

## 3. Results

**Figure 1:**
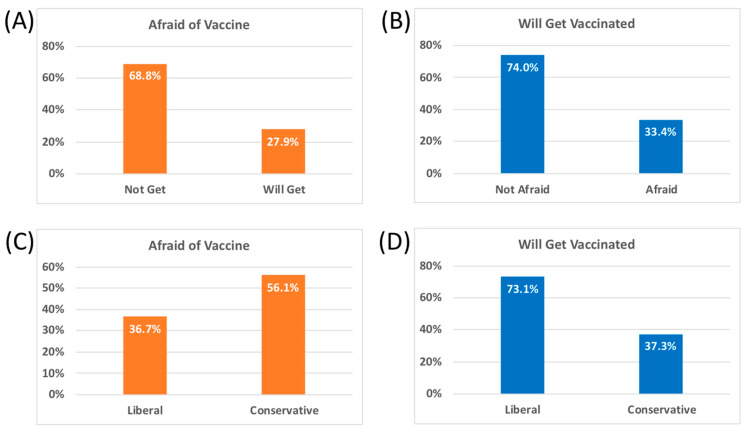
Opinions about the COVID-19 vaccine (*n* = 1017). (**A**) Proportion of individuals who report being afraid of the vaccine based on whether they indicate they will or will not get the vaccine. (**B**) Proportion of individuals willing to get vaccinated based on whether they endorsed fear of the vaccine. (**C**) Proportion of individuals reporting fear of the vaccine based on political ideology. (**D**) Proportion of individuals indicating they are willing to get the vaccine based on political ideology.

Overall, 46.2% of the sample indicated some fear of the vaccine (i.e., endorsed ≤ 3 on Item 1), and 55.3% indicated some willingness to get vaccinated immediately (i.e., endorsed ≥ 4 on Item 2). Importantly, of those who reported that they were afraid to get the vaccine, only 33.4% indicated that they would take the vaccine when offered, *χ*^2^(1) = 168.84, *p* = 1.32^−38^. Conversely, of those who indicated that they would agree to take the vaccine as soon as it is offered, 27.9% also endorsed that they were also afraid of taking the vaccine Figure 1A, B. These findings suggest that while a slight majority of individuals were willing to take the COVID-19 vaccine, there was lingering apprehension among a substantial segment of the population and this fear was associated with some reluctance to be vaccinated. A simple zero order correlation between fear of the vaccine and willingness to get the vaccine suggested a negative association (r = −0.606, *p* = 3.65^−103^). While highly significant, this suggests that that only 37% of the variance is shared between these variables, leaving another 63% unaccounted for.

To identify factors associated with fear of the vaccine and willingness to be vaccinated, we first calculated zero-order correlations between 13 potential predictor variables and the two outcome variables (see Table 2). After Bonferroni correction for multiple comparisons, conservative political ideology was the most highly correlated with both vaccine fear and intent to avoid receiving the vaccine. To further identify the variables with the greatest independent predictive power in the presence of other variables, we entered the 13 independent variables listed in Table 2 into two stepwise multiple linear regressions to predict fear of, and willingness to receive, the COVID-19 vaccine when available. Table 3 presents the relevant data and outcomes of the stepwise multiple regression analyses. First, being afraid to take the vaccine was significantly predicted by a linear combination of greater political conservatism, female sex, non-white ethnicity, greater generalized anxiety, lower annual income, and lower formal education (adjusted *R*^2^ = 0.184, *p* = 7.62^−43^). On the other hand, willingness to take the vaccine when offered was significantly predicted by a linear combination of greater political liberalism, greater fear of contracting COVID-19, male sex, higher formal education, higher annual income, lower psychological resilience, older age, white ethnicity, and having been previously diagnosed with COVID-19 (adjusted *R*^2^ = 0.349, *p* = 1.05^−89^). Being at high risk for COVID-19, or meeting screening criteria for depression, anxiety, or PTSD, however, were unrelated to willingness to take the vaccine in any regression models. Thus, political ideology appeared to be the most reliable predictor of vaccine fear and hesitancy. This is apparent in Figure 1C, as a greater proportion of those reporting conservative political ideology were likely to report fear of the vaccine, *χ*^2^(1) = 30.31, *p* = 3.68^−8^, while these same individuals were also much less willing to receive the vaccine when offered Figure 1D, *χ*^2^(1) = 106.50, *p* = 5.72^−25^ compared to those with a liberal ideology.

## 4. Discussion

As of December 2020, two approved vaccines had been shown to be highly effective and safe for the vast majority of the population [15]. We found that, during the week of the initial vaccine release in the U.S., just over half of the sampled individuals intended to get a COVID-19 vaccination as soon as possible. In other words, a nontrivial proportion still reported misgivings and intended to avoid accepting vaccination when offered, consistent with other recent data [16,17]. Remarkably, our findings suggest these concerns seemed to be dictated to a great extent by non-medical factors such as political ideology, and to a modest extent by factors such as sex, education, income, and ethnicity. Intention to get vaccinated was also predicted by the magnitude of fear of the novel coronavirus. However, fear of the vaccine itself and vaccine hesitancy were generally unrelated to psychiatric factors such as generalized anxiety, PTSD, or depression. These findings are consistent with an emerging pattern within the research literature suggesting that vaccine hesitancy for COVID-19 is largely dictated by sociological factors including political worldview and demographics [3,4,16,17,18,19].

In our analyses, we separately examined the worldview and demographic contributions to fear of the vaccine and willingness to get the vaccine. First, we found that fear of the vaccine only accounted for just over one third of the variance in vaccine hesitancy, suggesting that it is being driven primarily by factors other than vaccine fear. Interestingly, worldview and demographics accounted for nearly twice as much of the variance in vaccine hesitancy than for fear of the vaccine, suggesting that they are being driven by different combinations of factors. This makes sense, as the question about fear of the vaccine focuses primarily on an emotional reaction, while the question about intent to be vaccinated focuses on a behavioral decision. We found that worldview and demographics played only a modest, though significant role in the emotional response to the vaccine, but were highly associated with actual intent to engage in the behavior to seek out and obtain the vaccine. This is a potentially useful finding with regard to forthcoming approaches to the national vaccination campaign. Negative emotional reactions such as fear and anxiety are often automatically triggered and difficult to change due to reinforcement by avoidance [20]. Further, considerable evidence suggests that fear appeals tend to be relatively ineffective at producing attitude/behavior change [21]. Conversely, behavior and cognitions are often more amenable to modification through reasoning [22], particularly when the approach evokes positive emotions [23]. One implication of this finding is that information campaigns that align with individual socio-historical background and culture may be able to inspire behavioral action despite emotional reticence [24].

The present data suggest that the major contributor to vaccine hesitancy during the first month of vaccine rollout was political ideology. This suggests that without directly considering the socio-political worldview and demographic background of individuals, it will likely be difficult to engage a significant proportion of the population in the vaccination process. Recent evidence suggests that vaccination campaigns may be most effective if they address individual “local” concerns and are strategically communicated on an individual level [2]. While more research will be needed, we propose that local vaccine campaigns that focus on addressing individual socio-political worldview perspectives may be an important avenue for minimizing vaccine hesitancy. As political ideology was the strongest predictor of vaccine hesitancy, we suggest that it may be most helpful for frontline healthcare providers to discuss the vaccine with their hesitant patients within the framework of the patient’s worldview. For instance, for those patients whose worldview includes more liberal political values, it may be helpful for front line healthcare providers to discuss the vaccine from the perspective of its ability to protect the most vulnerable, ensure healthcare equality for all, and counter social injustices and healthcare disparities associated with the pandemic. For those patients who view the world from a more politically conservative perspective, we posit that it may be helpful for healthcare providers to frame widespread vaccination as a way to get people back to work, bolster the economy, protect individual freedoms, strengthen national security, and eventually reduce the need for social distancing and mask wearing. It is likely that strategically framing the benefits of the vaccine when communicating with individual patients may be a key factor in garnering collective national support for an effective vaccination campaign. This is likely best accomplished by frontline healthcare workers, as recent evidence suggests that information about vaccines may be most effective for minority individuals when given by a trusted healthcare provider [25]. Respected community leaders within particularly vulnerable populations (e.g., faith leaders, trusted local spokespersons) can help spread the message and bring transparency about the vaccine, its necessity, efficacy, and potential effects [26]. Given the unfortunate history of social injustices, many minority groups have legitimate reasons to be suspicious of largescale health efforts directed at their communities [27]. Given the urgency of the pandemic and the need for widespread vaccination, the potential utility of incorporating socio-political worldview into vaccine discussions is a topic in need of additional research.

Of course, it is important to acknowledge that these data were collected using the MTurk online data collection platform, which has a number of strengths and weaknesses. First, it is important to note that the vast majority of studies that have used MTurk have found it to be a valid and reliable method for collecting a variety of forms of psychological and health-related data [7,28], with most comparisons showing virtually no meaningful differences with data collected during in-person laboratory experiments [7], through campus surveys, or professional online survey services [29]. In fact, evidence suggests that participants on MTurk tend to provide better attention to instructions and sustained task performance than undergraduate student samples commonly used in much psychological research [8]. For some types of research, MTurk samples have been found to be more representative of attitudes than nationally representative panels [30]. During the course of the COVID-19 pandemic, we have collected data from multiple similar sized samples in the past that have all provided reliable data that are closely matched with other recent findings about psychological and behavioral health status [31,32,33,34,35,36]. For political ideology, as assessed here, data from MTurk surveys have been shown to be highly reliable and consistent with nationally representative samples [37]. Based on the previously described attention checks and screening measures employed, as well as the extensive literature suggesting that MTurk typically provides as good, or better, data than obtained in most face-to-face or local survey methods, we believe the present data are likely of high quality and reliability.

Despite the aforementioned strengths, it is important to acknowledge critical limitations to the current sampling approach. First, we did not collect a purely random sampling of individuals from the entire U.S. population. MTurk provides a limited sample frame of registered individuals who meet specific criteria, and may be subject to self-selection bias. MTurk workers must sign up for the platform and must also select to participate in the survey, and the present data need to be interpreted within that light. This is an important issue, as some have recently raised concerns regarding the generalizability of MTurk data to the larger U.S. population [9,10]. For instance, the demographics of MTurk samples, such as age, sex, and ethnicity have been found to differ from large nationally representative surveys in some studies [9,10]. Clearly, MTurk studies will not always provide data that are consistently matched with nationwide demographics, and MTurk survey data cannot be uncritically assumed to be directly representative of the entire U.S. population. While our sample included individuals from 48 states in the U.S., proportional to their populations, and did not differ from the census data in terms of majority/minority status, and our results are well-aligned with those of several other recently published studies [3,4,16,17,18,19], we also found that our sample included a greater proportion of females and was a few years younger than the U.S. population on average. We believe that caution is always warranted when generalizing from survey outcomes obtained from online platforms such as MTurk. Further research with nationally representative sampling is required to confirm the generalizability of the present findings.

## 5. Conclusions

In conclusion, our findings suggest that fears of the COVID-19 vaccine and willingness to get inoculated were highly associated with political worldview and demographic factors. In particular, vaccine hesitancy was predicted by greater political conservativism, low fear of COVID-19, female sex, lower education, age and income, and non-white ethnicity. Vaccine hesitancy continues to be a significant concern during the COVID-19 pandemic and needs to be addressed through multiple avenues based on scientific evidence. The most effective strategies will likely involve strategic framing of the vaccine in a manner congruent with individual worldview and demographic concerns at the local community level.

## Figures and Tables

**Table 1:** Demographic Characteristics and Scores on Survey Items.

Characteristic	Total Sample (N = 1017)
**Age—years**	37.0 ± 12.2
**Female sex—number (%)**	594 (58.4)
**Please Education—years**	15.1 ± 2.2
**Ethnicity—number (%)**	
** White**	770 (75.7)
** Black/African American**	113 (11.1)
** Hispanic/Latino**	47 (4.7)
** Asian**	55 (5.4)
** Native American/American Indian/Alaska Native**	7 (0.7)
** Native Hawaiian/Pacific Islander**	3 (0.3)
** Other**	22 (2.2)
** Prefer not to answer**	0 (0.0)
**Income—number (%)**	
** ≤USD 10,000**	63 (6.2)
** USD 10,001–USD 25,000**	126 (12.4)
** USD 25,001–USD 50,000**	275 (27.0)
** USD 50,001–USD 75,000**	196 (19.3)
** USD 75,001–USD 100,000**	156 (15.3)
** USD 100,001–USD 150,000**	126 (12.4)
** USD 150,001–USD 200,000**	42 (4.1)
** ≥USD 200,001**	33 (3.2)
**Political Ideology (1 = strongly liberal; 7 = strongly conservative)**	3.43 ± 1.90
**“Have you been formally diagnosed with COVID-19?” (yes)—number (%)**	60 (5.9)
**“Are you considered to be in a “high-risk” group for COVID-19” (yes)—number (%)**	317 (31.2)
**“I am gravely afraid of catching the COVID-19 virus” (1 = totally disagree; 7 = totally agree)**	4.27 ± 1.88
**“I’m afraid to get the new COVID-19 vaccine” (1 = totally disagree; 7 = totally agree)**	3.89 ± 1.96
**“I will get the new COVID-19 vaccine as soon as it is offered to me (1 = totally disagree; 7 = totally agree)**	4.49 ± 1.18
**GAD-7 (Generalized Anxiety Disorder scale-7)**	7.27 ± 6.03
**PHQ-9 (Patient Health Questionnaire-9)**	8.59 ± 6.70
**PC-PTSD (Primary Care Posttraumatic Stress Disorder)**	1.12 ± 1.48
**CD-RISC (Connor-Davidson Resilience Scale)**	64.25 ± 17.45

**Table 2:** Zero-order correlations between the 13 predictor variables and the vaccine items.

	Predictor Variables												
Dependent Variable	Political Ideology (Conservative)	Sex (Female)	Annual Income	Race/Ethnicity (White)	GAD-7 Anxiety	PHQ-9 Depression	PHQ-9 PTSD	I Am Gravely Afraid of Catching the COVID-19 Virus	CD-RISC	Dx with COVID-19 (Yes)	High Risk for COVID-19 (Yes)	Age	Education
“I’m afraid to get the new COVID-19 vaccine”	0.283 *	0.218 *	−0.162 *	−0.07	0.113 *	0.119 *	0.098 *	−0.038	0.029	0.018	0.06	−0.03	−0.182 *
“I will get the new COVID-19 vaccine as soon as it is offered to me”	−0.448 *	−0.114 *	0.10 *	0.019	0.076	0.048	0.060	0.414 *	−0.136 *	0.047	0.024	0.032	0.227 *

* *p* < 0.05, Bonferroni corrected.

**Table 3:** Results of Stepwise Multiple Linear Regression for Each Vaccine Item.

Vaccine Outcome Variable	*R*	*Adjusted R* ^2^	*F*	*p*-Value	Predictor Variables	*β*	t	*p*-Value	Partial *r*
“I’m afraid to get the new COVID-19 vaccine”	0.434	0.184	39.08	7.62^−43^	Political Ideology (conservative)	0.317	10.836	5.83^−26^	0.323
					Sex (Female)	0.197	6.785	1.97^−11^	0.209
					Race/Ethnicity (White)	−0.121	−4.226	0.0000	−0.132
					GAD-7 Generalized Anxiety	0.112	3.769	0.0002	0.118
					Annual Income	−0.105	−3.389	0.0010	−0.106
					Education (years)	−0.087	−2.84	0.0050	−0.089
“I will get the new COVID-19 vaccine as soon as it is offered to me”	0.596	0.349	61.52	1.04^−89^	Political Ideology (conservative)	−0.343	−12.028	3.11^−31^	−0.354
					“I’m gravely afraid of catching the COVID-19 virus” (yes)	0.308	10.985	1.36^−26^	0.327
					Education (years)	0.126	4.595	0.000005	0.143
					Sex (Female)	−0.145	−5.582	3.06^−8^	−0.173
					Annual Income	0.102	3.69	0.0002	0.116
					CD-RISC	−0.091	−3.464	0.001	−0.109
					Age	0.081	3.037	0.002	0.095
					Race/Ethnicity (White)	0.068	2.622	0.009	0.082
					Dx with COVID-19 (yes)	0.053	2.047	0.041	0.064

## Data Availability

Data available upon request to the corresponding author.
